# Correlates of depressive symptoms in urban middle-aged and elderly Lithuanians

**DOI:** 10.1007/s00127-014-0833-0

**Published:** 2014-02-26

**Authors:** Laura Sapranaviciute-Zabazlajeva, Regina Reklaitiene, Abdonas Tamosiunas, Migle Baceviciene, Dalia Virviciute, Anne Peasey

**Affiliations:** 1 Laboratory of Population Studies, Institute of Cardiology, Lithuanian University of Health Sciences, Medical Academy, Sukileliu 17, 50140 Kaunas, Lithuania; 2 Institute of Epidemiology and Health Care, University College London, London, UK

**Keywords:** Depressive symptoms, Health status, Life-style factors, Middle-aged and elderly Lithuanian adults

## Abstract

**Purpose:**

The study aimed to examine the prevalence of depressive symptoms and their correlates in urban middle-aged and elderly Lithuanian adults.

**Methods:**

Data from the survey was collected within the framework of the international project HAPIEE (Health, Alcohol and Psychosocial Factors in Eastern Europe). A random sample of 7,115 individuals aged 45–72 years was screened in 2006–2008.

**Results:**

Depressive symptoms were differently associated with independent variables by sex. In men, deprivation (OR 1.85, 95 % CI 1.54–2.17), being divorced (OR 2.34, 95 % CI 1.61–3.39) or widowed (OR 3.64, 95 % CI 2.40–5.52), physical inactivity (OR 1.30, 95 % CI 1.02–1.65), having a history of spine and joint disease (OR 1.72, 95 % CI 1.36–2.17), average perceived health (OR 2.14, 95 % CI 1.55–2.95), poor perceived health (OR 5.13, 95 % CI 3.39–7.76), average quality of life (OR 2.0, 95 % CI 1.55–2.95), or poor quality of life (OR 8.86, 95 % CI 5.19–15.13) were significantly associated with depressive symptoms. In women, deprivation (OR 1.28, 95 % CI 1.15–1.43), being widowed (OR 1.52, 95 % CI 1.23–1.88), mean dose of alcohol per occasion 40–79.9 g (OR 1.65, 95 % CI 1.18–2.30) and more than 80 g (OR 2.09, 95 % CI 1.14–3.82), physical inactivity in leisure time (OR 1.27, 95 % CI 1.04–1.57), having a history of spine and joint disease (OR 1.26, 95 % CI 1.06–1.51), average perceived health (OR 2.56, 95 % CI 1.89–2.72), poor perceived health (OR 5.07, 95 % CI 3.62–7.11), average quality of life (OR 2.27, 95 % CI 1.89–2.72), or poor quality of life (OR 7.21, 95 % CI 4.73–11.00) were significantly associated with depressive symptoms.

**Conclusions:**

Health status and lifestyle factors are associated with depressive symptoms. Associations between depressive symptoms and long-term health problems are partially mediated by self-rated quality of life and self-rated health.

## Introduction

The prevalence of depressive symptoms within the elderly population is quite high [1, 2]. Moreover, depressive symptoms in elderly adults tend to coincide with physical illness [3]. Higher levels of depressive symptoms often predict mortality in elderly people. This association is being explained by the comorbidity of physical illness [4]. However, some population-based studies show that depressive symptoms predict mortality even when co-occurrence of physical illness is being controlled [5].

Previous studies have revealed many correlates of depressive symptoms, the most commonly known being sex. Previous studies reveal that the prevalence of depressive symptoms is higher in women compared to men [1, 6, 7]. Marital status is also important—marriage works as a preventative factor for depressive symptoms, especially for men [4]. Depressive symptoms in Eastern Europe are also associated with current socioeconomic status measured by education level, deprivation, and crowding [8]. An especially high prevalence of depressive symptoms can be found within deprived populations [9].

Depressive symptoms are closely correlated with quality of life [10–12]. It is difficult to affirm cause and effect; however, some explanations have been suggested. The first is that participants with depressive symptoms perceive their quality of life as lower because of their lowered mood [13]. Also, older age with increased prevalence of chronic health problems is associated with lower quality of life [14]. Thus it might be that participants, because of long-term health problems, perceive their quality of life as lower.

In the elderly population, depressive symptoms are associated with self-evaluated health problems and a history of chronic diseases [6]. Previous studies have established that chronic diseases in older age lead to depressive symptoms [14, 15]. Depressive symptoms are more common in patients with asthma [16], respiratory diseases [17], cardiovascular diseases [5], and diabetes [18]. It is clear that depressive symptoms are associated with chronic illness. Associations become more severe with increasing quantity of chronic illness and chronicity; however, chronic illness does not explain all depressive symptoms [19]. Associations are not clear enough and previous studies found many differences due to sex or other covariates. Another hypothesis is that depressive symptoms are associated with a lower level of self-rated health because depressed patients subjectively perceive their health to be worse [13].

Depressive symptoms are also associated with health-related behavior. A great number of studies suggest that physical activity and exercise are inversely correlated with depressive symptoms [20]. The relationship between alcohol consumption and depressive symptoms is still unclear; some studies show that depressed participants tended to use alcohol more frequently [1], others posit that those two factors are not associated [21].

Many studies were done to explore the links between depressive symptoms, chronic illness, self-rated health or quality of life, lifestyle factors, and sociodemographic factors. Still, there is no clear answer about the correlates of depressive symptoms. Results differ because of population characteristics or the influence of other covariates. The purpose of this study is to examine the prevalence of depressive symptoms and their correlates in urban middle-aged and elderly Lithuanian adults.

## Methods

### Study sample

Our study presents data from the survey collected within the framework of the international project HAPIEE (Health, Alcohol and Psychosocial Factors in Eastern Europe) [22]. A random sample of 10,940 urban men and women from Kaunas aged 45–72 years, stratified by sex and age, were randomly selected from the Lithuanian population register. The response rate was 65 %, so 7,115 respondents participated in the survey in 2006–2008. Data of 86 male and 82 female participants were deleted from the analysis because of the missing data. Survey analyses data of 6,947 participants. Clinical and demographic characteristics of the sample are presented in Table 1. The study was approved by the Ethics Committee at University College London (UK) and by the Kaunas Regional Research Ethics Committee.

**Table 1:** Sample characteristics by gender (*N* = 6,947)

Sample characteristics	Men (*n* = 3,150)	Women (*n* = 3,797)
	Percentage or mean (SD)	Percentage or mean (SD)
Age	60.6 (7.6)	60.4 (7.6)
Education		
Primary	7.2	7.1
Vocational	28.7	35.6
Secondary	30.5	25.3
University	33.6	32.0
Marital status		
Married	85.0	57.1
Single	1.9	5.7
Divorced	7.7	16.0
Widowed	5.3	21.2
Deprivation (lower score more deprivation)	4.8 (0.5)	4.6 (0.7)
Crowding	2.7 (1.2)	2.4 (1.2)
Depressive symptoms (CES-D-10 ≥ 4)	15.6	29.9
Smoking		
Smokers	30.6	9.8
Ex-smokers	30.9	7.1
Non-smokers	38.4	83.1
Alcohol, mean dose per occasion (g)	156.3 (119.2)	81.7 (62.5)
Alcohol, annual intake (L)	1.4 (2.3)	2.4 (4.9)
Physical activity in leisure time (hours per week)	8.7 (6.3)	9.9 (5.9)
Body mass index (BMI) (kg/m^2^)	28.5 (4.6)	30.1 (5.8)
Chronic medical conditions		
Arterial hypertension	74.0	63.6
Diabetes	6.9	8.2
Ischemic heart disease	18.3	20.0
Long-term health problems	58.4	69.5
Chronic respiratory disease	14.4	18.1
Cancer	5.2	9.3
Gallbladder disease	9.6	16.3
Kidney stones	16.3	19.2
Asthma	3.0	4.9
Disease of spine and joins	45.2	60.0
Perceived health		
Very good, good	32.5	20.8
Average	56.1	61.4
Poor, very poor	11.4	17.8
Quality of life		
Very good, good	48.9	44.7
Average	47.7	50.3
Poor, very poor	3.4	5.0

### Measures

#### Depressive symptoms

Depressive symptoms were measured using the 10-item Center for Epidemiologic Studies Depression Scale (CES-D 10) [23]. It is a short self-report scale designed to measure depressive symptoms in epidemiological studies. Subjects were asked to evaluate the presence of ten depressive symptoms during the past week on a two-point scale: yes or no. Each symptom was scored from 1 (yes) to 0 (no), resulting in a total score of 0 to 10. On the basis of prior recommendations, subjects with a CES-D 10 score of 4 or more were classified as having depressive symptoms, and participants with a CES-D 10 score lower than 4 were classified as without depressive symptoms [24]. CES-D 10 scores should be carefully interpreted as an expression of perceived depressive symptoms, not as a clinical diagnosis [6]; specially trained personnel filled in the questionnaires with the respondents.

#### Sociodemographic and lifestyle factors

The standard questionnaire included questions regarding the respondent’s sociodemographic variables and lifestyle factors. Sample characteristics are presented in Table 1. Participants were asked about their education and marital status. Five categories of marital status (married, cohabiting, single, widowed, divorced) and five categories of education (university, college, vocational, primary, secondary) were listed in the questionnaire. As the cohabiting group was relatively small in the analysis it was combined with the married group. The college group was also combined with the university group. A deprivation score was assessed through questions about how often the person had difficulties in buying enough food or clothes and paying bills for housing, heating, and electricity. A higher deprivation score means a lower level of deprivation. Deprivation score ranged from 1 to 5, with the average value being 4.7 ± 0.6. Assessment of crowding was based on the number of inhabitants living in the respondent’s household. Smoking habits were assessed according to current smoking status. Annual alcohol intake was measured by asking participants how much wine, beer, and spirits they have had during the last 12 months. Also, alcohol consumption was measured by asking participants what the largest amount of alcohol they had on a single occasion during last month was. Physical activity was determined by the mean length of time spent per week during leisure time in winter and summer for walking, moderate and hard work like gardening, and other physical activities. The respondents were categorized into two groups according to their physical activity in leisure time: active in leisure time (10 h or more) and inactive in leisure time (<10 h). The respondents were also categorized into the following groups according to their self-rated health and quality of life: very good, good, average, poor, and very poor.

#### Chronic medical conditions

The survey also included a list of ten chronic medical conditions, namely chronic respiratory disease, cancer, gallbladder disease, kidney stones, asthma, disease of spine and joins, ischemic heart disease (IHD), diabetes, hypertension, and other long-term health problems. Participants were listed as having chronic respiratory disease, cancer, gallbladder disease, asthma, kidney stones, disease of the spine and joins, or long-term health problems if he/she had ever been diagnosed and hospitalized because of this disease. IHD was determined through two procedures—first, documented history of myocardial infarction (MI) and (or) ischemic changes on ECG coded by the Minnesota codes (MC) 1–1 or 1–2 [25]; second, angina pectoris was defined by the G. Rose questionnaire (without MI and (or) MC 1–1 or 1–2; 3) [26]; ECG findings by MC 1–3, 4–1, 4–2, 4–3, 5–1, 5–2, 5–3, 6–1, 6–2, 7–1, 8–3 (without MI and (or) MC 1–1, 1–2 and without angina pectoris). Diabetes was determined according to the respondent’s answer to the question “Has a doctor ever told you that you have diabetes?” and/or fasting glucose level ≥7.8 mmol/L. Hypertension was defined as systolic blood pressure ≥140 mmHg and/or diastolic blood pressure ≥90 mmHg, or normal blood pressure (<140/90 mmHg) if the person had taken antihypertensive drugs within the last 2 weeks.

#### Objective measurements

Blood pressure was measured three times, using an oscillometric device (Omron M5-I) after 5 min rest. The mean of three systolic and diastolic blood pressure tests was used. Waist circumference was measured by a standard meter within an accuracy of 0.5 cm. Body mass index (BMI) was calculated as weight (kilograms) divided by the square of height (meters). Biochemical analyses were done for participants on an empty stomach. The concentration of glucose in capillary blood was determined by an individual glucometer, Glucotrend [27].

### Statistical analysis

The prevalence of depressive symptoms was compared in gender groups via *χ* ^2^ tests. Owing to a significant difference in prevalence, means and distribution of variables according to the presence of depressive symptoms were examined in gender groups. Mean differences were tested via *t* test. Significant distribution differences were tested via *χ* ^2^ tests. For multivariate analysis we entered all variables which were significantly associated with depressive symptoms in univariate analysis. Multiple logistic regression analysis using the likelihood ratio criterion with SPSS version 13.4 software for Windows was used to analyze risk factors associated with depressive symptoms [28] at a significance level of 0.95 (*p* < 0.05).

## Results

### Demographic and clinical data

Table 1 shows demographic and clinical information for 6,947 survey respondents by gender. More than half were women aged 45–72 years, with a median age of 60.5. About 33 % had university degrees and almost 70 % were married or living with a partner at the time of the interview. The prevalence of depressive symptoms was 23.4 %. More than 60 % of the sample had never smoked. The highest occurrences of chronic medical conditions were arterial hypertension, disease of the spine and joints, and other long-term health problems; more than half of the participants had been diagnosed or hospitalized because of this condition. More than 26 % of participants evaluated their health as very good and good and almost half of the participants evaluated their quality of life likewise.

The prevalence of depressive symptoms was compared in male and female groups. There were more women with depressive symptoms compared with the male group—29.9 and 15.6 % respectively (*p* < 0.001). Owing to a significant difference in prevalence, means and distribution of variables according to the presence of depressive symptoms were examined in gender groups. Means and prevalence of different variables according to the presence of depressive symptoms are presented in Table 2. The mean and standard deviation are listed for continuous variables. Frequency and percentages are listed for categorical variables. Mean differences were tested via* t* test. Significant distribution differences were tested via *χ* ^2^ tests.

**Table 2:** Baseline participants characteristics by the presence of depressive symptoms

Variables	Men (*n* = 3,150)		Women (*n* = 3,797)	
	With depressive symptoms (*n* = 490)	Without depressive symptoms (*n* = 2,660)	With depressive symptoms (*n* = 1,134)	Without depressive symptoms (*n* = 2,663)
Depressive symptoms (CES-D-10 ≥ 4) (%)	15.6***	84.4	29.9	70.1
Age (%)				
45–54	24.5	24.0	22.3**	27.5
55–64	41.0	39.6	39.2	39.6
65–72	34.5	36.4	38.5**	32.9
Education (%)				
Primary	6.7	7.3	9.7***	5.8
Vocational	33.5**	27.5	36.8	35.1
Secondary	30.6	30.8	28.7**	24.0
University	29.2*	34.4	24.8***	35.1
Marital status (%)				
Married	70.2***	88.0	49.0***	60.7
Single	3.7***	1.5	5.3	6.0
Divorced	14.3***	6.4	18.3**	14.9
Widowed	11.8***	4.0	27.4***	18.4
Crowding (mean ± SD)	2.5 ± 1.1***	2.7 ± 1.2	2.3 ± 1.3*	2.4 ± 1.2
Deprivation (mean ± SD)	4.6 ± 0.7***	4.8 ± 0.5	4.4 ± 0.9***	4.7 ± 0.6
Smoking (%)				
Smokers	34.9*	29.8	10.1	9.6
Ex-smokers	32.2	30.6	7.8	6.9
Non-smokers	32.9**	39.5	82.1	83.5
Alcohol, mean dose per occasion (g) (mean ± SD)	41.4 ± 32.0	38.5 ± 29.2	21.5 ± 17.5**	19.9 ± 14.9
Alcohol, annual intake (L) (mean ± SD)	4.4 ± 8.1***	3.3 ± 5.3	0.5 ± 1.4	0.6 ± 1.1
Physical activity in leisure time (hours per week) (mean ± SD)	16.0 ± 13.1**	17.7 ± 12.5	17.6 ± 11.4***	20.7 ± 11.8
BMI (kg/m^2^) (mean ± SD)	28.2 ± 4.8	28.6 ± 4.6	30.6 ± 6.0***	29.8 ± 5.7
BMI (%)				
<25	26.9*	21.1	17.4	19.9
25–29.9	39.6*	44.8	33.9	36.1
≥30	33.5	33.9	48.6*	43.8
Arterial hypertension (%)	72.0	74.5	67.2**	62.0
Diabetes (%)	9.8***	6.4	10.6***	7.1
IHD (%)	24.9***	17.0	24.4***	18.2
Long-term health problems (%)	73.5***	55.8	80.2***	65.2
Chronic respiratory disease (%)	20.7***	13.3	22.7***	16.2
Cancer (%)	6.1	5.0	11.5***	8.6
Gallbladder disease (%)	12.2*	9.1	28.4***	22.6
Kidney stones (%)	20.2**	15.5	22.8***	17.6
Asthma (%)	4.1	2.9	6.4***	4.2
Disease of spine and joins (%)	59.2***	42.6	68.7***	56.5
Perceived health (%)				
Very good, good	12.6***	36.4	7.5***	26.6
Average	57.3	55.8	61.0	61.8
Poor, very poor	30.0***	7.8	31.5***	11.5
Quality of life (%)				
Very good, good	25.3***	53.4	24.7***	52.4
Average	62.2***	45.0	63.4***	44.7
Poor, very poor	12.0***	1.6	11.9***	2.0

Depressive symptoms were defined as CES-D 10 score 4–10, no depressive symptoms were defined as CES-D 10 score lower than 4. As compared to the group without depressive symptoms (distributions were compared using *χ* ^2^, means were compared using *t* test)

*SD* Standard deviation, *%* distribution of variables,* BMI* body mass index, *IHD* ischemic heart disease

* *p* < 0.05, ** *p* < 0.01, *** *p* < 0.001

### Univariate analysis

According to the results, age was related to depressive symptoms only in the female group. There were fewer women with depressive symptoms in the youngest age group and more women with depressive symptoms in the oldest age group. Education seemed to be associated with depressive symptoms—the prevalence of depressive symptoms was higher in the group of males that had undertaken vocational education and lower in university-educated males. Amongst women, the prevalence of depressive symptoms was lower in the university group and higher in participants with only primary and secondary education. Marital status was associated with depressive symptoms—there were more participants with depressive symptoms in the divorced and widowed groups and less in the “married” group. Being single was associated with a higher prevalence of depressive symptoms only for men. Less crowded living conditions were also associated with more depressive symptoms. Participants with depressive symptoms had a higher deprivation score. Men with depressive symptoms were more often smokers than men without depressive symptoms. Higher alcohol consumption per occasion was associated with depressive symptoms in women, whereas higher alcohol consumption per year was connected to depressive symptoms in men. Participants with depressive symptoms undertook less physical activity. BMI was higher in women with depressive symptoms according to the mean differences. Women with depressive symptoms were more often obese than women without depressive symptoms. However, different connections in the male group were established. There were more men with a BMI lower than 25 and less overweight men (25 < BMI < 29.9) with depressive symptoms compared to men without depressive symptoms. Prevalence of arterial hypertension was higher in women with depressive symptoms. Prevalence of all medical conditions was higher in participants with depressive symptoms compared to people without them, but prevalence of cancer and asthma did not differ significantly in the male group according to the expression of depressive symptoms. In the univariate analysis, perceived health and quality of life were associated with depressive symptoms. Participants with depressive symptoms had lower self-perceived health and quality of life compared to people without depressive symptoms.

### Multivariate analysis

Further, variables significantly related to depressive symptoms in univariate analysis were put into the multivariate analysis. Table 3 shows the multivariable odds ratios (ORs) for depressive symptoms adjusted for age, education, crowding, deprivation, marital status, smoking status, mean dose of alcohol per occasion and annual intake, physical activity in leisure time, BMI, arterial hypertension, diabetes, IHD, long-term health problems, chronic respiratory disease, cancer, gallbladder disease, kidney stones, asthma, disease of the spine and joints. In both sexes, deprivation was associated with depressive symptoms. Participants with a higher score of deprivation had significantly higher odds of depressive symptoms compared to those with lower deprivation level. Marital status was also connected to depressive symptoms in multivariate analysis. Divorced and widowed men had significantly higher odds of having depressive symptoms compared to married ones. In women, only status of widowhood had increased odds of depressive symptoms comparing to marriage. Mean dose of alcohol per occasion was associated with depressive symptoms only in females. Women consuming higher doses of alcohol had higher odds of depressive symptoms. All chronic medical conditions were not significantly associated with depressive symptoms in multivariate analysis except disease of the spine and joints, which had increased odds of depressive symptoms, especially amongst males. Perceived health and quality of life had the biggest effect on the incidence of depressive symptoms. Poor perceived health increased the odds of depressive symptoms by about fivefold and poor quality of life by about eightfold compared to a “very good” and “good” quality of life and self-rated health status.

**Table 3:** Odds ratios (ORs) and 95 % confidence intervals (CI) for depressive symptoms

	Men OR (95 % CI)	Women OR (95 % CI)
Deprivation	1.85 (1.54–2.17)***	1.28 (1.15–1.43)***
Marital status		
Married	1.0	1.0
Single	n.s.	n.s.
Divorced	2.34 (1.61–3.39)***	n.s.
Widowed	3.64 (2.40–5.52)***	1.52 (1.23–1.88)***
Mean dose of alcohol (g)		
0–19.9	n.s.	1.0
20–39.9	n.s.	n.s.
40–79.9	n.s.	1.65 (1.18–2.30)**
80+	n.s.	2.09 (1.14–3.82)*
Physical activity		
Active in leisure time	1.0	1.0
Inactive in leisure time	1.30 (1.02–1.65)*	1.27 (1.04–1.57)*
Disease of spine and joins	1.72 (1.36–2.17)***	1.26 (1.06–1.51)*
Perceived health (%)		
Very good, good	1.0***	1.0***
Average	2.14 (1.55–2.95)***	2.56 (1.98–3.38)***
Poor, very poor	5.13 (3.39–7.76)***	5.07 (3.62–7.11)***
Quality of life (%)		
Very good, good	1.0***	1.0
Average	2.0 (1.55–2.95)***	2.27 (1.89–2.72)***
Poor, very poor	8.86 (5.19–15.13)***	7.21 (4.73–11.00)***

CES-D 10 score from 4 to 10. ORs are adjusted for age, education, crowding and deprivation score, marital status, smoking status, mean dose of alcohol per occasion and annual intake, physical activity in leisure time, BMI, arterial hypertension, diabetes, IHD, long-term health problems, chronic respiratory disease, cancer, gallbladder disease, kidney stones, asthma, disease of spine and joins, perceived health, quality of life

*n.s.* not significant

* *p* < 0.05, ** *p* < 0.01, *** *p* < 0.001

## Discussion

As was found in the findings above, the prevalence of depressive symptoms in the middle-aged and elderly population is quite high [1, 2]. In our sample it was 23.4 %. Comparatively, the prevalence of depressive symptoms amongst older adults in China ranges from 12.8 to 41.1 % [2]. In Eastern European countries like Russia, Poland, and the Czech Republic, the prevalence of depressive symptoms is about 20 % in men and 40 % in women [1]. Our results corroborated previous studies that conclude that the prevalence of depressive symptoms is higher in women (29.9 %) compared to men (15.6 %) [9], although this may be because women might be more willing to express their emotions and negative feelings. It is also hypothesized that women tend to show more depressive symptoms due to health problems as they might be more sensitive to them [29]. However, different studies show that men are more overwhelmed with health problems and limitations to everyday activity [30]. Other studies show that, with all covariates controlled for, sex is still effective as an important factor for depressive symptoms [9, 31].

Age was correlated with depressive symptoms only in univariate analysis. Women showed more depressive symptoms in the older age group than the younger one. However, when all covariates were controlled for, age lost its effect on depressive symptoms, similar to previous studies [2]. This observation might be explained by the fact that the prevalence of depressive symptoms is higher in older participants because they have more chronic health problems than younger adults [9].

Marital status was associated with depressive symptoms for both men and women. Univariate analysis established that there were more participants with depressive symptoms in the “single”, “divorced”, and “widowed” groups than the “married” one. Even in multivariate analysis, marital status was important. Widowed participants had increased odds of having a higher level of depressive symptoms. Divorced men also tended to have a higher prevalence of depressive symptoms. As in previous studies, marriage worked as a preventative factor compared to those who have been divorced or widowed, especially for men [4]. It is perhaps understandable that widowers tend to be more depressed because of feelings of sorrow or loneliness.

Previous research has established different links between crowding and depressive symptoms. In similar populations to Lithuania like those of Russia, Poland, and Czech Republic no significant associations were found [32]. However, a study performed in North Carolina, in the immigrant Latino population, connected depressive symptoms to crowding. More crowded housing was associated with depressive symptoms [33]. In contrast, in our study, participants who have had depressive symptoms tend to live in less crowded houses. However, this link is not significant in multivariate analysis when deprivation and marital status were controlled for. It might be that crowding in our research was more connected to an indicator of marital status and less to an indicator of deprivation, so it did not indicate socioeconomic status.

Deprivation increases the probability of having depressive symptoms. In multivariate analysis, when all the risk factors were controlled for, deprivation still increased the odds of having higher levels of depressive symptoms in both the male and female groups. It confirms previous study results that depressive symptoms are associated with poor economic status [2, 8, 9]. The authors explain that people with lower socioeconomic status have fewer financial possibilities to reach their desired goals and to realize their potential, which is a circumstance connected to their psychological well-being [34]. We also suggest that because of their financial difficulties, their ability to solve health-related problems is affected, which in turn increases their chance of expressing depressive symptoms.

Health-related risk factors were differently connected to depressive symptoms in the male and female groups. Overweight and obese women tended to have more depressive symptoms, whereas this difference was not established in the male group. As a previous study shows, obesity is a risk factor for depressive symptoms only in women. This is probably because overweight women are stigmatized more than men [21]. In the univariate analysis, being a smoker was associated with depressive symptoms only in the male group; however, the same study in Japan revealed that smoking is a risk factor for developing depressive symptoms in women, too [21]. Our results might differ because of a very low incidence of smoking amongst the women in our analyzed age group. Our results established that a bigger dose of alcohol is connected with depressive symptoms in women, although the study in Japan revealed that alcohol consumption is not correlated with depression [21]. In countries closer to Lithuania, such as Russia, Czech Republic, and Poland, similar results were found to those in our study [2]. Physical inactivity in leisure time also increased the odds of having depressive symptoms in both men and women, which is an association confirmed by previous studies [20, 35].

In univariate analysis, all chronic diseases were connected to having depressive symptoms. Arterial hypertension, diabetes, IHD, long-term health problems, chronic respiratory disease, cancer, gallbladder disease, kidney stones, asthma, and disease of the spine and joints were all linked to depressive symptoms. These results reaffirm previous findings that established a link between depression and problems such as asthma [16], respiratory diseases [17], cardiovascular diseases [5], and diabetes [18]. In our study, arterial hypertension and cancer were connected to depressive symptoms only in female participants. Another study also concluded that amongst many chronic diseases, only cancer and hypertension were not linked to depressive symptoms in the study sample [3].

Our study showed that even controlling for sociodemographic factors and long-term health problems, a low quality of life was strongly associated with depressive symptoms, as shown in previous studies [12]. Health problems alone cannot explain association between quality of life and depressive symptoms. Although all chronic conditions were related with depressive symptoms in the univariate analysis, when controlling for self-rated quality of life and physical health associations, they lost their significance. Similar results were found in a study of the elderly Chinese population. In the bivariate analysis, depressive symptoms were associated with a higher number of chronic diseases and lower self-rated physical health. However, in multivariate analysis, only self-rated health was still a significant factor, which indicates that chronic disease might be a covariant of physical health and less relevant to depressive symptoms [2]. Authors of another study posit that multimorbidity and depressive symptoms are partially mediated by self-rated quality of life and self-rated health [3]. In conjunction with previous results, our study shows that for further studies and clinical implications, the main focus should be a general association of depressive symptoms and multiple chronic health conditions and not searching for specific links. The main target of further studies should be to understand the whole picture of interactions between depressive symptoms, self-rated health, and physical health status.

Although this study focused on many health indicators, lifestyle factors, and sociodemographic factors, and although the sample was representative, it has some limitations also. We used a self-reporting questionnaire to evaluate depressive symptoms, lifestyle factors, and health status, so mood could well have influenced the answers of participants at the time of filling out the questionnaire. As depressive symptoms were measured according to the participants’ perceptions during the last week, stressful life events could have an influence on it also. Moreover, the data was collected in 2006 to 2008—this limitation should be taken into account by the reader, even if it is unlikely that the association between health status and depressive symptoms has since changed.

## Conclusions

Corresponding to previous studies on the topic, sex was a very important factor in associations of depressive symptoms, health status, and lifestyle factors in middle-aged and elderly adults [21]. Preventive efforts should consider depressive symptoms’ associations with poor physical health, quality of life, physical inactivity, alcohol consumption, poor economic status, and take sex into account. The results of this study indicate that more attention should be given to deprived and lonely adults suffering from poor health. It also suggests a possible mediation between depressive symptoms and long-term health problems by self-rated health and quality of life. Further longitudinal studies are needed to analyze interactions between depressive symptoms, self-rated health, and physical health status.
